# Effectiveness of multidisciplinary intervention on blood pressure control in primary health care: a randomized clinical trial

**DOI:** 10.1186/s12913-016-1703-0

**Published:** 2016-08-31

**Authors:** Regina Kuhmmer, Rosmeri Kuhmmer Lazzaretti, Cátia Moreira Guterres, Fabiana Viegas Raimundo, Leni Everson Araújo Leite, Tássia Scholante Delabary, Suhelen Caon, Gisele Alsina Nader Bastos, Carisi Anne Polanczyk

**Affiliations:** 1 Institute for Education and Research, Hospital Moinhos de Vento, Rua Ramiro Barcelos 910, Bloco D, Porto Alegre, RS 90035-001 Brazil; 2 Graduate Program in Cardiology and Cardiovascular Science, Universidade Federal do Rio Grande do Sul. National Institute for Health Technology Assessment - IATS/CNPq, Porto Alegre, Brazil; 3 Department of Public Health, Universidade Federal de Ciências da Saúde de Porto Alegre, Porto Alegre, Brazil

**Keywords:** Hypertension, Public health, Multidisciplinary program, Systolic blood pressure, Diastolic blood pressure, Diabetes Mellitus

## Abstract

**Background:**

Hypertension is a public health problem and a major risk factor for cardiovascular disease. The purpose of this study is to compare the effectiveness of a multidisciplinary program based on group and individual care versus group-only care, to promote blood pressure control in hypertensive patients in primary health care.

**Methods:**

Randomized controlled clinical trial. The study was conducted within the primary health care, in two units of the Family Health Strategy, covering 11,000 individuals, in Porto Alegre, Brazil. Two hundred and 56 patients, older than 40 years old and with uncontrolled hypertension, systolic blood pressure (BP) ≥140 mmHg and/or diastolic BP ≥90 mmHg or ≥130 mmHg and/or diastolic BP ≥80 mmHg for individuals with diabetes. Eligible patients were randomly assigned to a health care program aiming for blood pressure control, with the multidisciplinary program group or with the multidisciplinary program plus personalized care group. Primary outcome measures were reduction in systolic BP from baseline to 6 months. Secondary measures included proportion of patients with systolic or diastolic BP controlled. Student *t* test, Pearson’s chi-squared test, Fisher’s exact test, Mann-Whitney *U* test, Wilcoxon signed-ranks test and generalized estimating equation (GEE) model were used in the analysis.

**Results:**

The baseline characteristics of participants were similar between groups. After 6 months of follow-up, systolic BP decreased markedly in both groups (Δ - 11.8 mmHg [SD, 20.2] in the multidisciplinary program group and Δ - 12.9 mmHg [SD, 19.2] in the personalized care group; *p* < 0.001). Similarly, we noted a significant change in diastolic BP over time in both groups (Δ - 8.1 mmHg [SD, 10.8] in the multidisciplinary program group and Δ - 7.0 mmHg [SD, 11.5] in the personalized care group; *p* < 0.001).

**Conclusions:**

The study demonstrates similar effectiveness of a group intervention in comparison to a personalized education program in hypertension patients to achieve BP control. These findings indicate that the intervention can be for all hypertensive patients assisted in primary health care.

**Trial registration:**

ClinicalTrials.gov IdentifierNCT01696318 (May 2013).

## Background

Hypertension is a public health problem throughout the world, affecting more than one billion people [1, 2]. The estimated prevalence in developing countries is 40 %, in comparison to 35 % in developed nations [3]. Studies indicate that reductions of 10 mmHg to 12 mmHg in systolic blood pressure (BP) or 5 mmHg to 6 mmHg in diastolic BP, or both, lower the risk of stroke by 35 to 40 %, cardiovascular death by 20 to 25 %, coronary heart disease by 14 to 16 %, and heart failure by 50 % [4–11].

In spite of well-established benefits of low BP, and the existence of several national and international guidelines on diagnostic and management of hypertension, control remains poor [12]. Canada has the lowest prevalence of hypertension at 19 %, followed by England and the United States of America (USA) at about 30 % each. However, only 34 % had BP under 140/90 mmHg in England, compared with 50 % in the USA and 66 % in Canada [13]. These numbers are even worse in developing countries, where hypertension prevalence is higher. In Brazil, approximately 30 % of adults are hypertensive [14], and BP control rates are even lower, ranging from 57.6 % to around 10 % [15, 16].

Health promotion programs related mainly to medication adherence, diet, physical activity and smoking habit have been introduced in different countries for prevention and control of cardiovascular risk factors [17]. Regarding the prevention of cardiovascular disease (CVD) in Brazil, the importance of public policies directed at nutrition and physical activity has been highlighted in the last few years. The National Diet and Nutrition Policy and the adoption of the Global Strategy on Healthy Eating, Physical Exercise and Health, of the World Health Organization (WHO) are examples of public policies, which include recommendations regarding healthy eating as a way to control and prevent CVD in primary health care (PHC) level [17, 18].

The Family Health Program and the Support Nucleus for the Family Healthcare (multidisciplinary program) are priority strategies of the Ministry of Health in order to organize PHC in Brazil [19]. It is believed that these strategies are adequate models for addressing CVD by means of prevention and health promotion, aimed to change the behavior and living habits of individuals, without losing sight of the interactions in collective and social spheres [20, 21]. However, no study has evaluated the effects of these programs in chronic disease patients, especially compared with other valid approaches. In this context, the objective of this study was to evaluate the effectiveness of a multidisciplinary program in PHC to promote BP control in hypertensive patients.

## Methods

### Study design and setting

The study was designed as a randomized, controlled clinical trial. It was conducted in two units under the Family Health Strategy (FHS), in Restinga and Extreme-South districts of Porto Alegre, Southern Brazil. These FHS units cover 11,000 individuals and Restinga is a low-income district, with approximately 100,000 people and a Human Development Index (HDI) of 0,700–0,799 [22].

### Participants

Patients were eligible for inclusion in the study if they were older than 40 years old, with previous hypertension diagnosis, and BP levels above recommended, measured by nurse technicians in screening in the FHS unit. Uncontrolled BP was defined as systolic BP ≥140 mmHg and /or diastolic BP ≥90 mmHg or ≥130 mmHg and /or diastolic BP ≥80 mmHg for individuals with diabetes [12, 23]. Institutionalized patients, with mental illnesses or disabling chronic illnesses, patients who were exclusively assisted by health insurance and those with a life expectancy of less than a year were not included in the study. Also, none of the participants could have been involved in a physical training program or a lifestyle change for 6 months prior to the study.

### Screening and recruitment

All patients who were assisted by a Family Health Team (FHT) with uncontrolled BP from May to July 2013 were invited to participate in the study. Eligible participants were identified through active screening by a research assistant. Three BP measures were performed according to the study protocol, using an appropriately sized cuff and a calibrated automated device with memory for storage of measurements (Omron HEM - 742 INT IntelliSense; Omron Healthcare) in the sitting position, after five minutes of rest, with no less than 1 min between measurements. The average of the second and third measures was used [12, 23].

### Interventions

#### Health professionals education

Health care professionals in the FHT (physicians, nurses, social workers) were invited to receive training, aiming to standardized care. The first training, which lasted 90 min, was performed by a cardiologist who focused on standard treatment algorithms for hypertension care and management, which were based on the Seventh Report of the Joint National Committee on Prevention, Detection, Evaluation, and Treatment of High Blood Pressure Guidelines (JNC 7) and the VI Brazilian Guidelines on Hypertension [12, 23].

Another training, which involved all professionals in the FHT, was conducted by a pharmacist, a dietitian and a physical educator who addressed no pharmacologic components (diet, exercise, weight loss, smoking cessation, and other recommendations) and pharmacologic interventions (prescription of drugs provided by the Unified Health System - UHS, and low-cost).

Manuals for health education were designed, based on evidence, for the four main areas of interest: pharmaceutical care (prepared considering the availability of drugs in public pharmacies), nutrition, physical activity, and strategies to be adopted in groups. These manuals were created in order to assist professionals during the study period, and also for the FHT to continue the personalized assistance with patients.

#### Patients education baseline

All patients included in the study were invited to participate in educational health workshops, with a dietitian, a physical educator, a pharmacist, and at least one member of the FHT. In these workshops, all participants were oriented to BP control, and received guidance on the benefits of having a healthy life and the deleterious effects of hypertension. The importance of achieving BP targets and adhering to medication was emphasized. After this activity, patients were randomized to the multidisciplinary program in the family healthcare group or multidisciplinary program plus personalized care group.

#### Multidisciplinary program for the family healthcare group

Multidisciplinary healthcare teams were established by Ordinance of the Ministry of Health, Decree No. 154 of January 24, 2008, under the name of the Support Nucleus for the Family Healthcare [19]. According to the Ministry, these programs should be performed by teams composed of different health care professionals, to support and to work in partnership with FHT with a focus on health practices in territories under their responsibility. The team who participated in this study consisted of a physical educator, a pharmacist and a dietitian, with the goal to promote adherence to drug treatment, to plan a balanced diet and to encourage the practice of physical activity. The activities offered by the multidisciplinary program were of standard care, and for this study the multidisciplinary program was considered the control group.

The multidisciplinary program group participated in monthly health education instruction and engaged in physical activity twice a week. Health education workshops were conducted in different locations for each of the geographical areas, to facilitate participants’ access, in places such as churches, halls, parks, schools and the participants’ residence.

The workshops covered topics related to hypertension through lectures and interactive dynamics, using posters, pictures, videos and practical demonstrations (concept, risk factors and treatment); physical activity (benefits and importance of weight reduction or maintenance, as well as improvement in quality of life and daily activities performance, they were also trained to perform the physical activities at home); medication adherence and dietary measures (consumption of fat, sugar and salt, recommendation and dangers of excessive consumption, and incentive to consume fruits and vegetables). Different kinds of fruits and vegetables were taken to the statement of recommended serving size and description of their beneficial health properties was made available. The participants were provided with systematic but flexible guidance, according to the needs and financial conditions of each of them.

A physical educator, in the presence of a pharmacist or a dietitian, and a member of the FHT, conducted a twice-a-week aerobic exercise training. The participants’ heart rate and BP were always measured at the beginning and the end of the activity. Each exercise session comprised an initial 5-min warm up, 50-min aerobic exercises and a final 5-min cool down phase. The physical activities also had a playful and relaxing profile.

#### Multidisciplinary program plus personalized care group

In addition to the group activities offered by the multidisciplinary program, the personalized care group (intervention group) also received referral to visit a dietitian and a clinical pharmacist with focus on hypertension control. A dietary approach was planned according to nutritional needs, socioeconomic status, and individual dietary habits. A 24-h dietary recall (24 h-DR) was applied in the 1st, 3rd and 6th month for assessment of dietary habits.

Counseling from the pharmacist included information about proper medication administration, side effects, and disease education. Pharmacists also reviewed patients’ medications and prescriptions by completing medication reconciliation; identifying duplicate, unnecessary, or incomplete therapy; checking for drug interactions; verifying patients’ formulary drug coverage and medications availability; and ensuring prescription completeness. To minimize variability during the counseling process, a standardized checklist was developed outlining the topics to be covered during a session, and standardized patient education leaflets were used. In order to identify the number of times each drug had to be taken, medication reconciliation was used, which included the names of all the drugs prescribed, with figures corresponding to the moment in which it had to be taken each time. The same illustrative figure was placed in the box of the product.

Patients were encouraged to check BP at home, and an automatic digital arm pressure monitor with memory for storage of measurements (Omron®, model HEM 742I) was provided twice, during the study period. During the appointment with the pharmacist, patients were trained and instructed to check BP twice a day for five days [24]. For individuals with diabetes, glucometers and test strips (Accu-Check®) for glucose control were provided during the entire study period.

#### Measurement and data collection

The primary outcome at 6th month was systolic BP reduction from baseline to the last follow-up visit. The secondary outcome was diastolic BP reduction from baseline to the last follow-up and the proportion of participants with controlled BP, <140 mmHg for systolic BP or <90 mmHg for diastolic BP and <130 mmHg for systolic BP or diastolic BP <80 mmHg for individuals with diabetes [12].

Other important patient data included demographic (gender, age, race and marital status), socioeconomic (education level and social class, according to the classification of the Brazilian Association of Research Companies - BARC [25]), and behavioral variables (smoking, alcohol consumption and physical activity). The Alcohol Use Disorders Identification Test (AUDIT) was applied to assess alcohol consumption, and the International Physical Activity Questionnaire (IPAQ) was used to evaluate the level of physical activity [26–28]. Height, weight, waist circumference and hip circumference were measured and the body mass index (BMI) was calculated [29].

The medication adherence was measured using two validated questionnaires in Portuguese: the Morisky-Green Test with four questions, and the Brief Medication Questionnaire (BMQ) with eleven questions [30–32]. The Charlson Comorbidity Index (CCI) was used to assess the presence of comorbidities. The absolute number for each condition identified was considered [33]. The Biochemical profile was assessed only at the end of the study, and included measurements of total cholesterol, high-density lipoprotein cholesterol (HDL), triglycerides, and low-density lipoprotein cholesterol (LDL), fasting blood glucose (FBG) and glycated hemoglobin (HbA1c) levels for individuals with diabetes, all with a 12-h fast. LDL-C was calculated using the Friedewald formula for those with triglycerides levels < 400 mg/dL [34].

For assessment of global cardiovascular risk we used the Framingham Risk Score (FRS) for nondiabetic individuals and the UKPDS Risk Engine (United Kingdom Prospective Diabetes Study) for individuals with diabetes [35, 36]. According to the FRS, patients were classified as low risk <10 %, intermediate risk 10–20 % and high risk > 20 % probability of cardiovascular event in 10 years.

#### Randomization

The randomization sequence was computer-generated and was assigned by a member of the study team who was blind to patient assignment until the intervention. Block randomization was used, with random block sizes of four, six and eight, in order to ensure similar size among the groups. During the study period, patients and health team members were aware of allocated groups. The randomization list was concealed in a central office.

#### Statistical analysis

A sample of 127 participants in each trial group (overall of 254) was planned, with an 80 % power, and a *p*-value of 0.05, to detect a reduction of 7.2 mmHg in systolic BP with standard deviation (SD) 20.45 mmHg in the intervention group and SD 20.26 mmHg in the control group [37]. Continuous variables are expressed as mean and SD or median interval interquartile (IQR), and categorical variables are expressed as proportions.

The groups were compared by means of the Student *t* test for continuous variables, and the Pearson’s chi-squared test or the Fisher’s exact test for categorical variables. The Mann-Whitney *U* test was used in between-group comparisons for variables not normally distributed. The Wilcoxon signed-ranks test for paired samples was used in within-group comparisons. Continuous variables taken at different time intervals were compared by generalized estimating equation model (GEE) to evaluate the effect of group allocation, adjusting for time effect (group * time). The variables were treated as normal distribution, with a connection identity function. The working correlation matrix used was unstructured and robust estimator covariance matrix. For significant effects was used post-hoc Bonferroni. Analysis of variance for linear trend was used to compare the reduction of BP in different levels of physical activity and medication adherence. Control for confounding factors regarding the reduction of BP levels was performed by multivariate linear regression analysis. For evaluation of medication adherence, the categories of BMQ were grouped into: high adherence and probably high adherence, and probably low adherence and low adherence. *P* values < 0.05 were considered statistically significant. All analyses were completed using the Statistical Package for the Social Sciences (version 20.0, SPSS, Chicago, Illinois).

## Results

### Patients

**Fig. 1:**
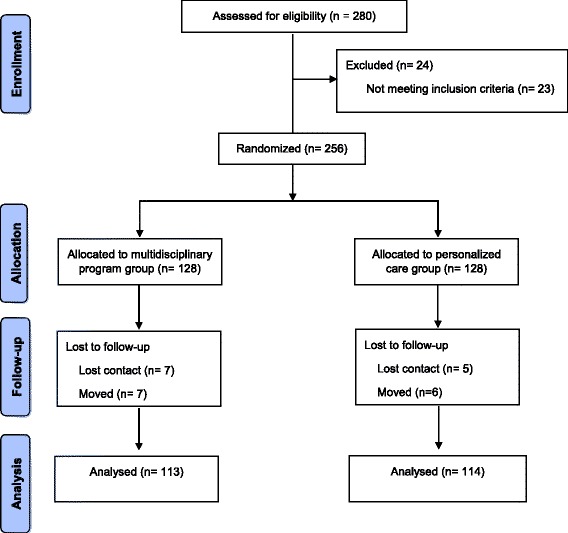
Study flow diagram

The study was conducted from July to December 2013. Among the 280 screened individuals, 256 (91 %) were considered eligible and were included in the study, 128 were randomly assigned to the multidisciplinary program group, and 128 to the personalized care group (Fig. 1). At the 6th month of the study, 16 patients were excluded from the multidisciplinary program group, due to the following reasons: five lost contact, six of them changed their addresses and three died, and 15 patients were excluded from the personalized care group: seven lost contact, seven changed their addresses and one died. The causes of death were acute myocardial infarction (AMI), stroke and gunshot in the multidisciplinary program group and AMI in the personalized care group.

### Baseline characteristics

Patients in each study group had similar baseline characteristics in respect to age, gender, education, BMI, smoking status, prevalence, baseline blood pressure and presence of chronic illness, with the exception of congestive heart failure history, more frequent in the control group. Most patients in the study (84 % for multidisciplinary program group and 83 % for personalized care group) had at least score ≥1 comorbidity of the Charlson Index; and 23 % of the patients in the multidisciplinary program group and 27 % in the personalized care group had diabetes (Table 1).

**Table 1:** Sociodemographic, clinical and behavioral characteristics of participants

Characteristic	Group		
	Multidisciplinary Program	Personalized Care	*p*-value
	*n* = 128	*n* = 128	
Gender, male	40 (31)	35 (27)	0.583^a^
Age, years	60 ± 11	59 ± 10	0.527^b^
Race			
White	98 (77)	95 (74)	0.652^a^
Yellow	17 (13)	22 (17)	
Black	13 (10)	11 (9)	
Years of education completed			
0 – 4	62 (48)	59 (46)	0.410^a^
5 – 8	41 (32)	50 (39)	
≥ 9	25 (20)	19 (15)	
Marital status			
Married	74 (58)	78 (61)	0.364^a^
Single	14 (11)	8 (6)	
Separated	13 (10)	19 (15)	
Widowed	27 (21)	23 (18)	
Social class, BARC			
High, A/B	37 (29)	33 (26)	0.277^a^
Medium, C	62 (48)	74 (58)	
Low, D/E	29 (23)	21 (16)	
BMI, Kg/m^2^	30 ± 0.5	30 ± 0.5	0.784^b^
Blood pressure, mmHg			
Systolic	156 ± 2	158 ± 2	0.437^b^
Diastolic	89 ± 1	90 ± 1	0.633^b^
Comorbidity Index			
CCI	2 (1–3)	2 (1–4)	0.704^c^
CCI-Y	4 (2–5)	3 (2–5)	0.951^c^
CIC-Y, survival 10 years	53 (11–85)	56 (16–82)	0.843^c^
Comorbidity			
Previous myocardial infarction	16 (13)	10 (8)	0.214^a^
Heart failure	22 (17)	11 (9)	0.040^a^
Peripheral vascular disease	13 (10)	13 (10)	1.000^a^
Stroke	18 (14)	17 (13)	0.856^a^
Diabetes Mellitus	32 (23)	34 (27)	0.564^a^
COPD	9 (7)	5 (4)	0.272^a^
Connective tissue disease	57 (45)	69 (54)	0.134^a^
Gastric ulcers	24 (19)	24 (19)	1.000^a^
Liver disease	12 (9)	13 (10)	1.000^a^
Renal disease	17 (13)	20 (16)	0.594^a^
Cancer	10 (8)	5 (4)	0.237^a^
Smoking status			
Never	50 (43)	52 (47)	0.563^a^
Current	25 (21)	20 (18)	
Past	42 (36)	38 (35)	

*BARC* Brazilian association of research companies, *BMI* Body mass index, *CCI* Charlson comorbidity index, *CCI-Y* Charlson comorbidity-year index, *COPD* Chronic obstructive pulmonary disease. Values expressed n (%), mean and standard error (SE) or median and interquartile range (IQR)

^a^Person chi-square test or Fisher’s exact test

^b^Student’s *t*-test

^c^Mann-Whitney *U* test

### Blood pressure and hypertension control

During the study period, systolic BP decreased in both treatment groups, Δ - 11.8 mmHg (SD, 20.2) in the multidisciplinary program group, (*p* < 0.001) and Δ - 12.9 mmHg (SD, 19.2) in the personalized care group, (*p* < 0.001), with no significance between the groups, *p* = 0.60. Similarly, a significant change in diastolic BP was also noted in respect to time in both groups, Δ - 8.1 mmHg (SD, 10.8) in the multidisciplinary program group (*p* < 0.001) and Δ - 7.0 mmHg (SD, 11.5) in the personalized care group (*p* < 0.001), and no significance between groups was observed, *p* = 0.36 (Table 2). In the secondary outcome analysis, a significant increase in the proportion of patients with controlled systolic and diastolic BP (*p* < 0.001) was detected, between baseline and 6-months in both groups (Table 3). In addition, the relative risk of not achieving the systolic BP target in individuals with diabetes was 1.64 times higher than in nondiabetics (95 % CI 1.33 to 2.03) and 2.87 in diastolic BP (95 % CI 1. 87 to 4.41).

**Table 2:** Blood pressure and clinical measurements from baseline to end of study

Variable	Group		*p*-value		
	Multidisciplinary Program	Personalized Care	Group	Time	Interaction
	*n* = 113	*n* = 114			
Systolic BP, mmHg					
Baseline	156 ± 1.6 (128)	158 ± 1.8 (128)	0.575	<0.001	0.600
6 Months	144 ± 1.8 (114)	143 ± 1.8 (113)			
Δ	- 11.8 ± 20.2	- 12.9 ± 19.2			
Diastolic BP, mmHg					
Baseline	89 ± 1.02 (128)	90 ± 1.03 (128)	0.286	<0.001	0.365
6 Months	80 ± 1.03 (114)	82 ± 1.09 (113)			
Δ	- 8.10 ± 10.8	- 7.0 ± 11.5			
BMI, Kg/m^2^					
Baseline	29.9 ± 0.5 (128)	30.1 ± 0.5 (128)	0.745	0.454	0.859
6 Months	30 ± 0.5 (114)	30.2 ± 0.5 (113)			
Δ	0.08 ± 1.9	0.13 ± 2.9			
WC, cm					
Baseline	95.1 ± 1.7 (127)	96.1 ± 1.2 (126)	0.438	0.122	0.608
6 Months	95.6 ± 1 (113)	96.9 ± 1.1 (113)			
Δ	0.40 ± 5.9	0.82 ± 6.6			
WHR					
Baseline	0.91 ± 0 (127)	0.92 ± 0 (126)	0.482	0.098	0.482
6 Months	0.90 ± 0 (113)	0.91 ± 0 (113)			
Δ	- 0.012 ± 0.06	- 0.003 ± 0.88			
Appointments, number					
Baseline	3.82 ± 0.4	2.95 ± 0.2	0.258	<0.001	0.084
6 Months	1.9 ± 0.2	2.02 ± 0.2			
Δ	- 1.94 ± 0.4	- 0.93 ± 0.3			
Physical activity, min per week					
Baseline	87 ± 12	96 ± 14	0.114	<0.001	0.110
6 Months	188 ± 17	245 ± 27			
Δ	103 ± 15	151 ± 26			

*BP* Blood pressure, *BMI* Body mass index, *WC* Waist circumference, *WHR* Waist-to-hip ratio. Values expressed n (%), mean and standard error (SE), *GEE* Generalized estimating equationan overview of five randomized controlled

**Table 3:** Blood pressure control from baseline to end of study

Variables	Group							
	Multidisciplinary Program			Personalized Care			Between group	
	Baseline	6 Months	*p*-value	Baseline	6 Months	*p*-value	Baseline	6 Months
	*n* = 128	*n* = 113		*n* = 128	*n* = 114		*p*-value	
Systolic BP								
Nondiabetic	7 (9)	40 (49)	<0.001	5 (7)	44 (57)	<0.001	0.451	0.744
Diabetic	0 (0)	9 (27)	0.003	0 (0)	8 (22)	0.014		
Diastolic BP								
Nondiabetic	37 (47)	68 (86)	<0.001	36 (47)	62 (81)	<0.001	0.697	0.473
Diabetic	6 (18)	17 (52)	0.003	5 (14)	19 (53)	<0.001		

*BP* blood pressure. BP control defined as systolic BP <140 or diastolic BP 90 mmHg for nondiabetic patients and systolic BP level <130 mmHg or diastolic BP <80 mmHg for diabetic patients. Values expressed n (%). Person chi-square test

### Pharmacological treatment and medication adherence

The pharmacologic treatment during the study was similar for all drug classes. The number of antihypertensive medication prescribed was similar in both groups in the baseline (multidisciplinary program group 83 % vs. personalized care group 83 %, *p* = 0.74), and did not increase significantly over time (multidisciplinary program group 86 % vs. personalized care group 88 %, *p* = 0.85). Although subjects of both groups were prescribed more antihypertensive drugs, this increase was similar between groups (multidisciplinary program group *p* = 0.29 and personalized care group, *p* = 0.06) (Table 4). Both in the multidisciplinary program group and in the personalized care group there was a percentage of patients without drug prescription, and this condition did not change between and within groups at the end of the study, *p* = 0.98. Medication adherence measured by the Morisky-Green Test increased from 34 to 49 % in the multidisciplinary program group and from 35 to 55 % in the personalized care group. In relation to the BMQ test, the medication adherence increased from 35 to 68 % in the multidisciplinary program group and from 22 to 67 % in the personalized care group. There was no significant difference between groups in both tests (Table 5).

**Table 4:** Pharmacologic treatment

Variable	Multidisciplinary Program			Personalized Care			Between groups	
	Baseline	6 Months	*p*-value	Baseline	6 Months	*p*-value	Baseline	6 Months
	*n* = 128	*n* = 113		*n* = 128	*n* = 114		*p*-value	
Medication class								
Not prescription	5 (4)	9 (8)	0.289	13 (12)	10 (8)	0.453	0.084	0.984
Antihypertensives	94 (83)	98 (86)	0.289	94 (83)	99 (88)	0.063	0.743	0.845
Antidiabetic	29 (26)	30 (27)	1.000	31 (28)	34 (31)	0.508	0.388	0.558
Antidepressive agents	27 (24)	25 (22)	0.880	34 (30)	29 (26)	0.533	0.322	0.536
Other cardiovascular drugs	36 (32)	40 (35)	0.481	45 (40)	5 (40)	1.000	0.090	0.095
Pulmonary	5 (5)	6 (5)	1.000	6 (6)	7 (6)	1.000	0.769	0.784
Hypolipidemic agents	36 (32)	40 (35)	0.503	39 (35)	41 (37)	0.774	0.687	0.890
Antihypertensive drugs								
Diuretics	49 (44)	55 (49)	0.286	49 (44)	59 (53)	0.052	1.000	0.592
Beta blockers	31 (27)	38 (34)	0.118	35 (32)	39 (36)	0.424	0.342	1.000
ACE	66 (58)	66 (58)	1.000	63 (57)	63 (57)	1.000	0.704	0.789
ARBs	10 (9)	13 (12)	0.508	17 (16)	23 (21)	0.70	0.079	0.150
CCB	19 (17)	22 (20)	0.453	15 (14)	21 (19)	0.146	0.486	0.867

*ACE* Angiotensin-converting enzyme inhibitor, *ARBs* Angiotensin II Receptor Blockers, *CCB* Calcium channel blockers. Values expressed n (%). Person chi-square test

**Table 5:** Behavioral and medication adherence

Variable	Group							
	Multidisciplinary Program			Personalized Care			Between groups	
	Baseline	6 Months	*p*-value	Baseline	6 Months	*p*-value	Baseline	6 Months
	*n* = 128	*n* = 113		*n* = 128	*n* = 114		*p*-value	
IPAC								
Active	25 (22)	56 (49)	0.001	24 (21)	59 (52)	<0.001	0.930	0.750
Insufficient active	39 (34)	40 (35)		41 (36)	40 (35)			
Inactive	50 (44)	18 (16)		48 (43)	14 (12)			
Considers health								
Very bad	7 (6)	7 (6)	0.133	8 (7)	2 (2)	0.047	0.701	0.253
Bad	13 (11)	13 (11)		14 (12)	8 (7)			
Regular	55 (48)	44 (39)		46 (41)	42 (37)			
Good	38 (33)	45 (40)		42 (37)	53 (47)			
Very Good	1 (1)	5 (4)		3 (3)	8 (7)			
BP self-monitoring								
Every day	16 (14)	28 (25)	<0.001	13 (12)	21 (19)	<0.001	0.532	0.473
Once week	28 (25)	29 (25)		22 (20)	38 (34)			
Once month	19 (17)	42 (37)		20 (18)	36 (32)			
Rarely	49 (43)	13 (11)		57 (50)	17 (15)			
Never	2 (1.8)	2 (2)		1 (1)	1 (1)			
BMQ								
High adherence	35 (35)	69 (68)	<0.001	21 (22)	64 (67)	<0.001	0.093	0.996
Low adherence	66 (65)	32 (32)		75 (78)	32 (33)			
TMG								
Adherence	34 (34)	49 (49)	0.008	34 (35)	53 (55)	<0.001	0.635	0.810
Moderate Adherent	55 (55)	47 (47)		44 (46)	39 (41)			
Low adherence	12 (12)	5 (5)		18 (19)	4 (4)			
Improved self-esteem		90 (79)			102 (90)			<0.001

*IPAC* International Physical Activity Questionnaire*, BP* blood pressure, *BMQ* Brief Medication Questionnaire*, TMG* Test Morisky-Green*, AUDIT* Alcohol Use Disorder Identification Test. Values are n (%). Person chi-square test

### Physical activity

The amount of physical activity performed increased in the multidisciplinary program group and in the personalized care group; *p* < 0.001. In addition, the percentage of active people increased from 22 to 49 % in the multidisciplinary program group (*p* < 0.001), and from 21 to 52 % in the personalized care group (*p* < 0.001). However, there was no significant difference between groups. In the linear trend analysis, patients classified as active by IPAC had a greater BP control; systolic BP (*p* = 0.043), diastolic BP (*p* = 0.039), but when adjusted for gender and medication adherence, only medication adherence remained significant (*p* = 0.027).

### Anthropometric measures, biochemical profile and cardiovascular risk

Regarding anthropometric measures, no differences were observed between the groups in variables such as BMI, waist circumference (WC) and waist-to-hip ratio (WHR) within 6 months (Table 2). BP self-monitoring in both groups increased over time, *p* = 0.001, but there was no difference between groups, *p* = 0.473 (Table 4). The results of biochemical profile and of cardiovascular risk are shown on Table 6. Biochemical tests were not available for most patients at baseline; therefore, only 6-month values are presented. The groups did not significantly differ at the end of the study, regarding biochemical profile and cardiovascular risk, both in individuals with and without diabetes (Table 6). The percentage of smoking and alcohol consumption decreased in both groups, but it was not of significance (data not shown).

**Table 6:** Cardiovascular risk factors at the end of the study

Variable	Group		
	Multidisciplinary Program	Personalized Care	*p*-value
	*n* = 113	*n* = 114	
FBG, mg/dL			
Diabetic	137 ± 52 (32)	135 ± 36 (33)	0.905^a^
Nondiabetic	92 ± 16 (68)	93 ± 15 (67)	0.712^a^
HbA1c, %	6.6 ± 2 (32)	6.4 ± 1 (33)	0.634^a^
Total cholesterol, mg/dL	191 ± 47 (107)	198 ± 47 (104)	0.263^a^
HDL, mg/dL	45 ± 12 (107)	45 ± 12 (104)	0.916^a^
LDL, mg/dL	113 ± 41 (96)	119 ± 41 (98)	0.297^a^
Triglyceride, mg/dL	143 (113 – 190) (107)	143 (112 – 213) (104)	0.769^b^
Nondiabetic, 10 years FRS, n%			
Low	47 (63)	39 (55)	0.528^c^
Medium	19 (25)	24 (34)	
High	9 (12)	8 (11)	
Mean FRS	5 (2–14)	6 (3–14)	0.647^b^
Diabetic, UKPDS, risk 10 year, %			
CHD	12 (8 – 17) (32)	11 (6 – 19)	0.572^b^
Fatal CHD	7 (4–10) (32)	6 (3 – 13)	0.682^b^
Stroke	7 (5 – 12) (32)	5.5 (3 – 12)	0.993^b^
Fatal Stroke	1 (1 – 2) (32)	1 (1 – 2)	0.992^b^

*FBG* Fasting blood glucose, *HbA1c* Glycated hemoglobin, *HDL* High-density lipoprotein, *LDL* Low-density lipoprotein cholesterol, *FRS* Framingham risk score, *CVD* Cardiovascular disease. Values expressed n (%), mean and standard deviation (SD) or median and interquartile range (IQR)

^a^Student’s *t*-test

^b^Mann-Whitney *U* test

^c^Person chi-square test

### Dietary assessment

There were no statistically significant differences in total calories intake, macronutrients (carbohydrate, protein and total lipids), saturated fat acid (SFA), polyunsaturated fat acid (PFA) and monounsaturated fat acid (MFA) between groups after the intervention. Similarly, cholesterol, micronutrients (calcium, iron and potassium), fibers and sodium did not differ significantly. Regarding fruit ingestion, there was a significant higher consumption in the personalized care group (data not shown).

## Discussion

In this study we evaluated the effectiveness of a multidisciplinary program, suggested by the Ministry of Health, to promote BP control in hypertensive patients in PHC, in Brazil. The results of this study show that among adults with uncontrolled BP, an education strategy with multidisciplinary program alone or combined with personalized care in BP management reduced systolic and diastolic BP significantly and increased the proportion of patients with BP on target.

Our educational program resulted in 11.8 mmHg and 12.9 mmHg reductions in systolic BP in both multidisciplinary program group and personalized care groups, respectively. For nondiabetic individuals, rates of systolic BP under control were of 8.6 to 49.4 % in the multidisciplinary program group and of 6.6 to 57.1 % in the personalized care group. In individuals with diabetes, the systolic BP control was of 0 to 27 % in the multidisciplinary program group and of 0 to 22.2 % in the personalized care group. Our control rate was lower in individuals with diabetes than in nondiabetic individuals; however, literature data indicate that the rate control in these individuals could be as low as 3 %, similar to findings at the beginning of this study [38].

A systematic review of 24 observational studies including 47,964 individuals, with both hypertension and diabetes, reported that only 12 % (range 6 to 30 %) of participants had controlled BP [39].

Our study showed significant BP reductions in both groups without alterations on medications prescribed. There are some potential explanations for the good BP results, including BP goal reinforcement by the education program, medication adherence and lifestyle modifications, as well as regular physical activity practice. Increased adherence to medications probably played an important role in the results observed. In the study of Guiraro et al. [40], the treatment adherence measured by the Morisky-Green Test increased by 9.6 % in the intervention group and 8.8 % in the control group. The intervention consisted of personalized information by a trained nurse and written leaflets and the individuals allocated to the control group received the usual clinical care without any standardized intervention. In our study, there was a 15 % increase in the multidisciplinary program group and 20 % in the personalized care group.

Healthy life style, especially engagement in physical activities was highly emphasized, and also could have had some effect on BP control. The protocol required that both groups practiced physical activity twice a week and participated in health education workshops once a month. It was demonstrated in a prior study that more participants in the exercise group (56.7 %) than in the control group (35.5 %) attained adequate BP control (<140/90 mmHg) post-12-week interventions [41].

Although prior trials suggested that empowerment, constant feedback and individualized care are more effective in achieving medication and care adherence, in our study the combination of these elements in the personalized care did not translate into greater BP control than in the multidisciplinary program. This result suggests that the activities conducted in groups can be as effective as individual guidelines, if performed accordingly.

No reductions were observed in anthropometric measurements in our study. Similarly, in a cluster randomized controlled trial, Harris MF et al. [42], found a small weight reduction (1.06 kg) that was achieved only among those attending the intervention group, participating in the education program. Improved glycemic control reduced microvascular and macrovascular complications associated with type 2 diabetes mellitus. At the 6th month, we found an average of 6.6 mg/dL in the multidisciplinary program group and 6.4 mg/dL in the personalized care group, in HbA1c. However, the lack of baseline data precludes any conclusion regarding diabetes control in our population.

Self-care activities which help to control blood glucose levels and to avoid diabetes-related complications are important in diabetes treatment. Doucette et al. [43] observed, in a randomized clinical trial, that a pharmacist-provided diabetes care service led to significant improvement in self-management and self-care activities in individuals with diabetes. A main challenge for our study was to motivate individuals to participate in the activities proposed. Rates of attendance and completion of lifestyle programs are often poor and highly variable in general practice [44].

This study was unable to determine the variables that influenced the reduction of BP. However, it was designed to assess the effect of an education program to promote BP control in hypertensive individuals in PHC, not to determine the mechanisms by which a reduction in BP was achieved. It is believed that the benefit of an education program on lowering BP was due to a combination of strategies and not one isolated element, such as changes in lifestyle, physical activity and greater medication adherence [45].

Some caveats of this study ought to be considered. Firstly, variance in BP could have occurred because observations within groups may have correlated, that is, both groups were composed of people who visited the same places, lived in the same neighborhood or nearby, or may be more similar than those in different locations. Secondly, the values of biochemical tests were not available at baseline, and it was believed that these patients should have had several of these examinations in their routine, but they were not available. Moreover, the 24 h-DR was not performed at baseline in the multidisciplinary program group, only at the 6th months. This fact limits comparisons of groups at baseline and at 6 months and it cannot be stated that dietary data changed during the period.

## Conclusions

The study demonstrates the effectiveness of a multidisciplinary program intervention in BP control in the setting of primary care. The combination of personalized care, involving nutritionists and clinical pharmacists, did not translate into additional benefit of achieving BP control. These findings indicate that the model proposed for the Family Health Program by the Support Nucleus for the Family Healthcare (multidisciplinary program) must be considered for all hypertensive patients assisted in PHC. Other studies with longer period of follow up should be conducted to evaluate the impact of these interventions on clinical outcomes associated with biochemical profile and cardiovascular risk.

## Abbreviations

24 h-DR: 4-h dietary recall
AMI: Acute myocardial infarction
AUDIT: Alcohol use disorders identification test
BARC: Brazilian association of research companies
BMI: Body mass index
BMQ: Brief medication questionnaire
BP: Blood pressure
CCI: Charlson comorbidity index
CI: Confidence interval
CVD: Cardiovascular disease
CVD: Cardiovascular disease
FBG: Fasting blood glucose
FHS: Family health strategy
FHT: Family health team
FRS: Framingham risk score
GEE: Generalized estimating equation
HbA1c: Glycated hemoglobin
HDI: Human development index
HDL: High-density lipoprotein cholesterol
IPAC: International physical activity questionnaire
IQR: Interval interquartile
LDL: Low-density lipoprotein cholesterol
MFA: Monounsaturated fat acid
PFA: Polyunsaturated fat acid
PHC: Primary health care
SD: Standard deviation
SFA: Saturated fat acid
USA: United States of America
WC: Waist circumference
WHO: World Health Organization
WHR: Waist-to-hip ratio
